# Development of risk prediction models for depression combining genetic and early life risk factors

**DOI:** 10.3389/fnins.2023.1143496

**Published:** 2023-07-18

**Authors:** Tianyuan Lu, Patrícia Pelufo Silveira, Celia M. T. Greenwood

**Affiliations:** ^1^Lady Davis Institute for Medical Research, Jewish General Hospital, Montreal, QC, Canada; ^2^Department of Psychiatry, Faculty of Medicine, McGill University, Montreal, QC, Canada; ^3^Ludmer Centre for Neuroinformatics and Mental Health, Douglas Research Center, McGill University, Montreal, QC, Canada; ^4^Department of Epidemiology, Biostatistics and Occupational Health, McGill University, Montreal, QC, Canada; ^5^Department of Human Genetics, McGill University, Montreal, QC, Canada; ^6^Gerald Bronfman Department of Oncology, McGill University, Montreal, QC, Canada

**Keywords:** polygenic risk score, early life risk factors, depression, risk assessment, predictive modeling

## Abstract

**Background:**

Both genetic and early life risk factors play important roles in the pathogenesis and progression of adult depression. However, the interplay between these risk factors and their added value to risk prediction models have not been fully elucidated.

**Methods:**

Leveraging a meta-analysis of major depressive disorder genome-wide association studies (*N* = 45,591 cases and 97,674 controls), we developed and optimized a polygenic risk score for depression using LDpred in a model selection dataset from the UK Biobank (*N* = 130,092 European ancestry individuals). In a UK Biobank test dataset (*N* = 278,730 European ancestry individuals), we tested whether the polygenic risk score and early life risk factors were associated with each other and compared their associations with depression phenotypes. Finally, we conducted joint predictive modeling to combine this polygenic risk score with early life risk factors by stepwise regression, and assessed the model performance in identifying individuals at high risk of depression.

**Results:**

In the UK Biobank test dataset, the polygenic risk score for depression was moderately associated with multiple early life risk factors. For instance, a one standard deviation increase in the polygenic risk score was associated with 1.16-fold increased odds of frequent domestic violence (95% CI: 1.14–1.19) and 1.09-fold increased odds of not having access to medical care as a child (95% CI: 1.05–1.14). However, the polygenic risk score was more strongly associated with depression phenotypes than most early life risk factors. A joint predictive model integrating the polygenic risk score, early life risk factors, age and sex achieved an AUROC of 0.6766 for predicting strictly defined major depressive disorder, while a model without the polygenic risk score and a model without any early life risk factors had an AUROC of 0.6593 and 0.6318, respectively.

**Conclusion:**

We have developed a polygenic risk score to partly capture the genetic liability to depression. Although genetic and early life risk factors can be correlated, joint predictive models improved risk stratification despite limited improvement in magnitude, and may be explored as tools to better identify individuals at high risk of depression.

## Introduction

Depression is a common and serious illness that affects a large proportion of the population, is associated with an increased risk of suicide, and causes significant mortality, morbidity (Wong and Licinio, 2001; Hawton et al., 2013), and economic losses (Sartorius, 2001). Accurately identifying individuals at high risk may help to better design and target preventive interventions, thus reducing the socioeconomic burden (Djernes, 2006; King et al., 2008). However, depression is a highly heterogeneous disease with multifactorial causes and is often comorbid with other complex diseases, thus complicating risk prediction modeling (Kendler et al., 1993; Martin and Dahlen, 2005; Maier et al., 2015; Lu et al., 2023).

Early life stress, maladaptation, or dangerous experiences, such as domestic violence (Sternberg et al., 1993; Wiersma et al., 2009; Mandelli et al., 2015), have been widely recognized as risk factors for psychiatric diseases, high-risk behaviors, and other adverse health outcomes (Finkelhor et al., 2015; Metts et al., 2023). Children exposed to early life risk factors are at a substantially higher risk of developing adult depression, and this vulnerability can endure throughout their lifetime (Sadowski et al., 1999; McLaughlin, 2016). Several epidemiological studies have demonstrated that various measures of early life risk factors may predict future adulthood diagnoses of depression (Kessler and Magee, 1993; Felitti et al., 1998; Gibb et al., 2007; Bjorkenstam et al., 2017), as well as recurrence and chronicity of depression (Gilman et al., 2003). Evidently, unlike clinical risk factors from adulthood, exposure to early life risk factors usually precedes the onset of depression. This fact means that, for prediction modeling, reverse causation is unlikely for early life risk factors. Therefore, risk prediction models including early life factors may be particularly useful for designing preventive measures, although it remains challenging to comprehensively measure and integrate diverse risk factors.

Another important risk factor of depression is genetic predisposition (Wray et al., 2018; Howard et al., 2019; Giannakopoulou et al., 2021). Previous twin studies estimated that ~40% of the liability to major depressive disorder (MDD) could be attributed to genetic factors (Sullivan et al., 2000). Genome-wide association studies (GWASs) have revealed the polygenic architecture underlying MDD, wherein hundreds of genes harbor variants associated with the risk of MDD (Wray et al., 2018; Howard et al., 2019; Giannakopoulou et al., 2021). Such polygenic effects may be quantified by polygenic risk scores (PRSs), which aggregate the cumulative risk conferred by genetic variants throughout the genome. PRSs can be predictive of disease outcomes and have demonstrated potential clinical utility in population-level risk stratification (Inouye et al., 2018; Khera et al., 2018; Martin et al., 2019a; Lu et al., 2021a,b), risk factor identification (Lewis and Vassos, 2020; Lu et al., 2020a, 2022a), differential diagnosis (Sharp et al., 2019; Lu et al., 2020b, 2022b), treatment optimization (Damask et al., 2019; Marston et al., 2020), and possibly encouraging risk-reducing behaviors (McBride et al., 2010).

Although the importance of both genetic and early life risk factors in adult depression has been recognized, the interplay between these risk factors in altering risk predictions has not been fully elucidated. Notably, little attention has been paid to the potential added value derived from combining these two kinds of risk factors. Therefore, in this study, we first developed a PRS for depression based on a large-scale meta-analysis of MDD GWASs. We next assessed the association between this PRS and several early life risk factors of depression in the UK Biobank (Bycroft et al., 2018), as well as their associations with depression phenotypes. Finally, we harnessed the combined predictive power of genetic and early life risk factors by stepwise regression and thereby evaluated whether joint predictive models could improve the identification of at-risk individuals.

## Methods

### UK Biobank

Between 2006 and 2010, more than 500,000 individuals participated in the UK Biobank study (Bycroft et al., 2018). These participants were recruited at multiple assessment centers located in the United Kingdom. Upon recruitment, the participants provided demographic and health care information. Follow-up of the participants was conducted during repeat assessment visits, by online questionnaires or by surveying electronic health-related records. We leveraged the most recent data release that contained health-related records updated by March 2020.

Genome-wide genotyping was conducted using blood samples with the Applied Biosystem™ UK BiLEVE Axiom™ Array or UK Biobank Axiom™ Array, followed by imputation to the Haplotype Reference Consortium reference panel (Genomes Project Consortium et al., 2015). Genetic ancestry of each participant and their genetic relatedness were determined based on genotyped variants after quality control (data field 22,006), as described previously (Bycroft et al., 2018). Specifically, the UK Biobank inferred the genetic ancestry of each individual by matching clusters of the first six genetic principal components to self-reported ancestral groups. Kinship coefficients between each pair of individuals were calculated using KING (Manichaikul et al., 2010). In cases where two individuals were third-degree or closer relatives, one individual was excluded to form a subpopulation consisting of unrelated individuals. Due to the limited cross-ancestry portability of polygenic risk scores (Martin et al., 2017, 2019b) and small sample sizes of non-European ancestry populations in the UK Biobank, we restricted our analyses to 408,822 unrelated individuals (without third-degree or closer relationships) of European ancestry in this study, based on genetic ancestry assignment and genetic relatedness provided by the UK Biobank. We assigned these individuals into a model selection dataset (*N* = 130,092), consisting of individuals in the interim release of the UK Biobank data in 2015, and a test dataset (*N* = 278,730), consisting of individuals not in the interim release but in the full release in 2018.

### Definition of depression phenotypes

Following Howard et al. (2018), we defined three depression phenotypes: broad depression that included any possible mental health difficulties, probable MDD that included more severe symptoms of depression, and International Classifications of Diseases (ICD)-10-coded MDD that was based on physician-made diagnoses.

Specifically, cases of broad depression were defined as having sought treatment for nerves, anxiety, tension, or depression (based on data field 2090, “Have you ever seen a general practitioner (GP) for nerves, anxiety, tension or depression” and data field 2,100, “Have you ever seen a psychiatrist for nerves, anxiety, tension or depression” from questionnaire), or having an ICD-10 code of F32 (single episode depression), F33 (recurrent depression), F34 (persistent mood disorders), F38 (other mood disorders) or F39 (unspecified mood disorders) upon recruitment or during any of the follow-up visits. Controls were individuals who had never sought treatment for mental health difficulties and had none of the ICD-10 codes. Individuals who did not respond to the questionnaire, or answered “do not know” or “prefer not to answer” were considered as missing data.

Cases of probable MDD were defined as having sought treatment for nerves, anxiety, tension, or depression while having been depressed, unenthusiastic, or disinterested for at least 2 weeks (based on data field 4,598 “ever depressed for a whole week,” data field 4,609 “longest period of depression,” data field 4,631 “ever unenthusiastic/disinterested for a whole week,” and data field 5,375 “longest period of unenthusiasm/disinterest” from questionnaire), or having an ICD-10 code of F32, F33, F34, F38, or F39 upon recruitment or during any of the follow-up visits. Controls were individuals who had never been depressed, unenthusiastic, or disinterested for a whole week and had none of the ICD-10 codes. Individuals who did not meet the criteria for either cases or controls, as well as those who did not respond to the questionnaire, or answered “do not know” or “prefer not to answer” were considered as missing data.

Cases of ICD-10 MDD were defined as having an ICD-10 code of F32, F33, F34, F38, or F39 upon recruitment or during any of the follow-up visits. Controls were individuals who had none of the ICD-10 codes and were controls of probable MDD. Other individuals were considered as missing data.

### Definition of early life risk factors

We considered six possible early life risk factors of depression obtained from questionnaire: whether an individual was part of a multiple birth (data field 1,777), birthweight (data field 20,022), whether an individual was breastfed as a baby (data field 1,677), maternal smoking around birth (data field 1,787), domestic violence, and access to medical care. Extreme birthweight was defined as having a birthweight below the 10^th^ percentile or above the 90^th^ percentile. Individuals who responded “very often,” “often,” or “sometimes” to the question “Physically abused by family as a child” (data field 20,488) or the question “Sexually molested as a child” (data field 20,490) were considered as victims of frequent domestic violence; these individuals, together with those who responded “rarely” to either of these two questions were considered as victims of any domestic violence; Individuals who responded “never” to both of these two questions were considered as not being exposed to domestic violence as a child. Individuals who responded “very often,” “often,” or “sometimes” to the question “Someone to take to doctor when needed as a child” (data field 20,491) were considered as having access to medical care; those who responded “rarely” or “never” were considered as not having access to medical care. Individuals who did not respond to the questionnaire, or answered “do not know” or “prefer not to answer” were considered as missing data.

### Development of polygenic risk score

We leveraged the results of MDD GWAS meta-analyses performed by the PGC (Wray et al., 2018). We obtained the GWAS summary statistics from the meta-analysis which did not include the UK Biobank, based on a total of 45,591 cases and 97,674 controls, predominantly of European ancestry. Definition of MDD cases has been described previously (Wray et al., 2018), which included cohort-specific structured diagnostic interviews and electronic health-related records.

We adopted LDpred (Vilhjalmsson et al., 2015) to develop a PRS for MDD. LDpred operates on the GWAS summary statistics to incorporate genome-wide genetic variants into a predictive model (PRS), while accounting for linkage disequilibrium (LD) between genetic variants with an LD reference panel (Vilhjalmsson et al., 2015). We used HapMap3 variants (International HapMap Consortium et al., 2010) from a random subset of 5,000 European ancestry UK Biobank participants to compute the LD reference panel. We constructed eight candidate PRSs with a varying tuning parameter (p) determining the fraction of variants to be considered as causal: *p* = infinity (i.e., LDpred-inf), *p* = 1, *p* = 0.3, *p* = 0.1, *p* = 0.03, *p* = 0.01, *p* = 0.003, and *p* = 0.001. Genetic variants included in these PRSs were HapMap3 variants with a minor allele frequency > 0.001, an imputation quality score > 0.3, and a missing rate < 0.1 (Bycroft et al., 2018).

Next, in the model selection dataset, for each candidate PRS, we assessed its discriminative power in identifying individuals at high risk of broad depression, probable MDD, and ICD-10 MDD, respectively, by area under the receiver-operating characteristic curve (AUROC). For each depression phenotype, we selected the PRS with the highest AUROC as the optimized PRS for downstream analyses. We examined whether this PRS was normally distributed by Kolmogorov–Smirnov test.

### Association between polygenic risk score, early life risk factors, and depression phenotypes

In the test dataset, we first assessed the associations between the PRS and each of the early life risk factors by logistic regression. Chi-square tests were used to assess associations between each pair of early life risk factors, with the magnitudes of associations quantified by tetrachoric correlation (i.e., Pearson correlation for two binary variables).

We then tested for the association between each depression phenotype and the PRS, and between each depression phenotype and each of the early life risk factors by logistic regression. We also tested whether there existed interaction effects between the PRS and early life risk factors on depression phenotypes by multivariate logistic regression. Furthermore, we compared the magnitudes of the depression-PRS associations and the depression-early life risk factor associations. Specifically, when comparing to an early life risk factor affecting α % of the population, we binarized the population at the (100−α) -th percentile of the PRS, and assessed the disease risk in the high-risk group in contrast to the low-risk group by logistic regression. All logistic regression models adjusted for the fixed effects of age, age (Hawton et al., 2013), sex, recruitment center, genotyping array, and the first 10 genetic principal components to account for population stratification.

When testing for PRS-early life risk factor interaction effects, we further adjusted for the PRS-covariate interaction effects as well as early life risk factor-covariate interaction effects (Keller, 2014). However, it is important to note that these models included several correlated variables, which can result in increased multicollinearity. This, in turn, may lead to imprecise and unstable model estimates and an increased risk of overfitting. Therefore, these analyses should be interpreted as sensitivity analyses.

Benjamini-Hochberg-corrected *p*-values (false discovery rate, FDR) were calculated to adjust for the multiple testing conducted across all early life risk factors and three depression phenotypes.

### Joint predictive modeling using genetic and early life risk factors

To further improve risk prediction, we fitted a joint predictive model for each depression phenotype including the PRS, all early life risk factors, age, age (Hawton et al., 2013), and sex as predictors. We replaced the binary risk factors for low or high birthweight by birthweight and birthweight (Hawton et al., 2013), which improves the flexibility for modeling possible non-linear effects and reduces collinearity. We performed stepwise regression to reduce model redundancy due to correlation between risk factors, using the R package MASS with default settings (minimization of the Akaike information criterion). We termed the resulting model as the PRS + RF model.

In addition, we repeated the model fitting and variable selection, separately without the PRS, without any early life risk factors, and with only age, age (Hawton et al., 2013), and sex. We termed the resulting models as the RF model, the PRS model, and the baseline model, respectively.

The significance of each predictor in each of the final joint predictive models was determined based on multivariate regression including all retained predictors. To evaluate model consistency, we compared the coefficients of each predictor obtained from multivariate regression to those obtained from univariate regression using the same set of individuals included in the multivariate analysis.

### Assessment of predictive performance

In the test dataset, we evaluated the performance of the above joint predictive models in predicting the risk of respective disease. AUROC and area under the precision-recall curve (AUPRC) were calculated for each model and the corresponding depression phenotype using the R package PRROC. To evaluate whether the PRS + RF model had significantly better predictive performance than the RF model and the PRS model, we resampled individuals in the test dataset 1,000 times with replacement, and obtained 95% confidence intervals for AUROC and AUPRC. Bootstrap *p*-values were calculated based on the frequency at which the RF model or the PRS model achieved a higher AUROC or AUPRC across the 1,000 replicates. Moreover, we calculated net reclassification indices (NRI) at different cutoffs (50th, 80th, or 90th percentile of the predicted risk) for defining the high-risk group as well as integrated discrimination indices (IDI; Pencina et al., 2008) using the R package PredictABEL, where a positive NRI and a positive IDI would indicate improved discriminative power using the PRS + RF model over the RF model and the PRS model.

## Results

### Study design

**Figure 1:**
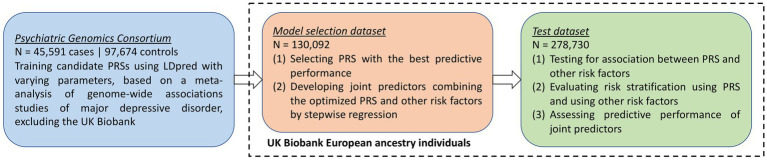
Overview of study design.

An overview of the study design is provided in Figure 1. In this study, we first developed candidate polygenic risk scores based on a meta-analysis of MDD GWASs conducted by the PGC (Wray et al., 2018; section Methods). We leveraged the UK Biobank resources to select the best-performing polygenic risk score as follows. The UK Biobank participants of European ancestry were assigned into a model selection dataset (N = 130,092) and a test dataset (*N* = 278,730). These two datasets had similar demographic characteristics (Table 1), with a mean age of 56.9 years (standard deviation, SD = 7.9 years in the model selection dataset and 8.0 years in the test dataset), and more females (52.8% in the model selection dataset and 54.6% in the test dataset) than males. After excluding individuals with missing data, maternal smoking around birth was the most prevalent early life risk factor among all risk factors investigated, affecting 32.6% of the individuals in the model selection dataset and 29.9% in the test dataset, while only 2.3% of the individuals in both datasets were part of a multiple birth (Table 1).

**Table 1:** Cohort characteristics.

	Model selection dataset (*N* = 130,092)	Missing data in model selection dataset (%)	Test dataset (*N* = 278,730)	Missing data in test dataset (%)
Mean age (year; SD)	56.9 (7.9)	0	56.9 (8.0)	0
Female (%)	68,720 (52.8)	0	152,322 (54.6)	0
Part of a multiple birth (%)	2,966 (2.3)	1,896 (1.5)	6,319 (2.3)	3,774 (1.4)
Mean birthweight (kg; SD)	3.3 (0.7)	55,972 (43.0)	3.3 (0.7)	119,101 (42.7)
Breastfeeding (%)	69,870 (71.0)	31,645 (24.3)	151,150 (71.3)	66,736 (23.9)
Maternal smoking (%)	36,459 (32.6)	18,285 (14.1)	71,728 (29.9)	38,876 (13.9)
Frequent domestic violence (%)	4,383 (10.8)	89,467 (68.8)	9,191 (10.2)	188,461 (67.6)
Any domestic violence	9,740 (24.0)	89,467 (68.8)	20,847 (23.1)	188,461 (67.6)
Access to medical care (%)	39,631 (97.2)	89,334 (68.7)	88,127 (97.4)	188,242 (67.5)
Broad depression^†^	47,949 (37.1)	711 (0.5)	101,559 (36.7)	1,628 (0.6)
Probable MDD^†^	18,690 (45.5)	89,021 (68.4)	39,066 (44.8)	191,561 (68.7)
ICD-10 MDD^†^	7,348 (24.8)	100,518 (77.3)	14,572 (23.4)	216,404 (77.6)

† Proportions of individuals with depression phenotypes do not represent prevalence of disease, because individuals with missing data were removed.

In the model selection dataset, the PRS developed with an LDpred parameter *p* = 0.03 (section Methods) achieved the highest AUROC in predicting all three depression phenotypes (Supplementary Figure S1). Therefore, this PRS was used for quantifying genetic predisposition toward depression phenotypes in all downstream analyses. The distribution of PRS did not differ from a normal distribution in the UK Biobank (Supplementary Figure S2).

We did not detect any significant interaction effect between the PRS and early life risk factors in the model selection dataset (value of *p* > 0.05; Supplementary Table S1), hence interaction effects were not evaluated in the test dataset and were not included for developing joint predictive models.

We jointly modeled all genetic and early life risk factors for predicting each of the three depression phenotypes. In all PRS + RF models, the PRS, maternal smoking around birth, frequent domestic violence, any domestic violence, and having access to medical care were retained as predictors after variable selection using stepwise regression (section Methods; Supplementary Table S2). Being breastfed as a child was retained in the PRS + RF models for predicting probable MDD and ICD-10 MDD, while being part of a multiple birth was only retained in the PRS + RF model for predicting ICD-10 MDD. None of the PRS + RF models included birthweight or birthweight (Hawton et al., 2013) due to the small magnitude of effect and correlation with other predictors. All model coefficients are provided in Supplementary Table S3. These coefficients obtained from multivariate regression were largely consistent with those obtained from univariate regression for each predictor based on the same set of individuals (section Methods; Supplementary Table S3).

### Polygenic risk score for depression was associated with early life risk factors

**Figure 2:**
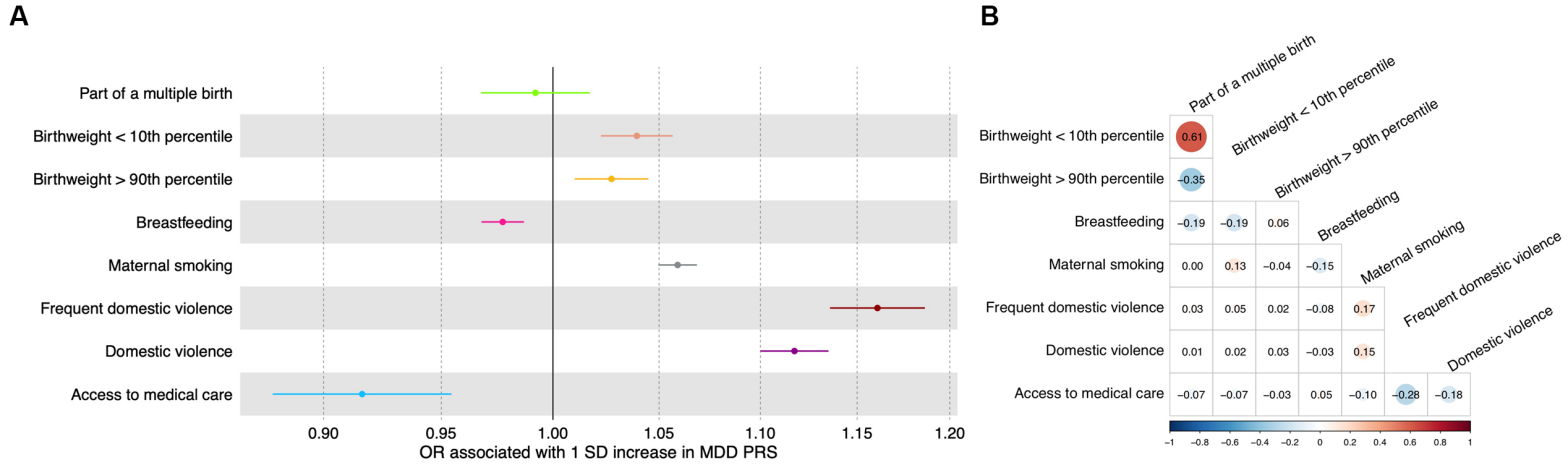
Association between genetic and early life risk factors. **(A)** Odds ratios of being exposed to early life risk factors associated with a one standard deviation increase in the PRS. Increased PRS was positively associated with not being breastfed as a baby and not having access to medical care. Error bars indicated 95% confidence intervals. Detailed test statistics are provided in Supplementary Table S1. **(B)** Correlation between early life risk factors. Tetrachoric correlation coefficients are indicated with size of dots corresponding to the magnitude of correlation. Chi-square test *p*-values are provided in Supplementary Table S2. Correlation between low birthweight and high birthweight and correlation between frequent domestic violence and any domestic violence were not calculated because these variables represent the same underlying risk factors. OR, odds ratio; SD, standard deviation.

In the test dataset, the PRS for depression was significantly associated with most early life risk factors investigated in this study. Specifically, a one SD increase in the PRS was significantly associated with frequent domestic violence as well as any domestic violence, with an odds ratio of 1.16 (95% CI: 1.14–1.19; value of *p* = 5.6 × 10^−41^; FDR = 2.2 × 10^−40^) and 1.12 (95% CI: 1.10–1.13; value of *p* = 6.1 × 10^−44^; FDR = 4.9 × 10^−43^; Figure 2A), respectively. A one SD increase in the PRS was marginally associated with increased odds of having a birthweight below the 10th percentile, having a birthweight above the 90th percentile, not being breastfed as a child, maternal smoking around birth, and not having access to medical care (Figure 2A; Supplementary Table S4), respectively. The PRS was not significantly associated with being part of a multiple birth (Figure 2A; Supplementary Table S4).

Meanwhile, as expected, the early life risk factors could be correlated with each other (Figure 2B; Supplementary Table S5), sometimes strongly. For instance, having access to medical care was negatively associated with frequent domestic violence (correlation coefficient = −0.28) and any domestic violence (correlation coefficient = −0.18; Figure 2B; Supplementary Table S5).

### Genetic and early life risk factors were associated with depression phenotypes

**Figure 3:**
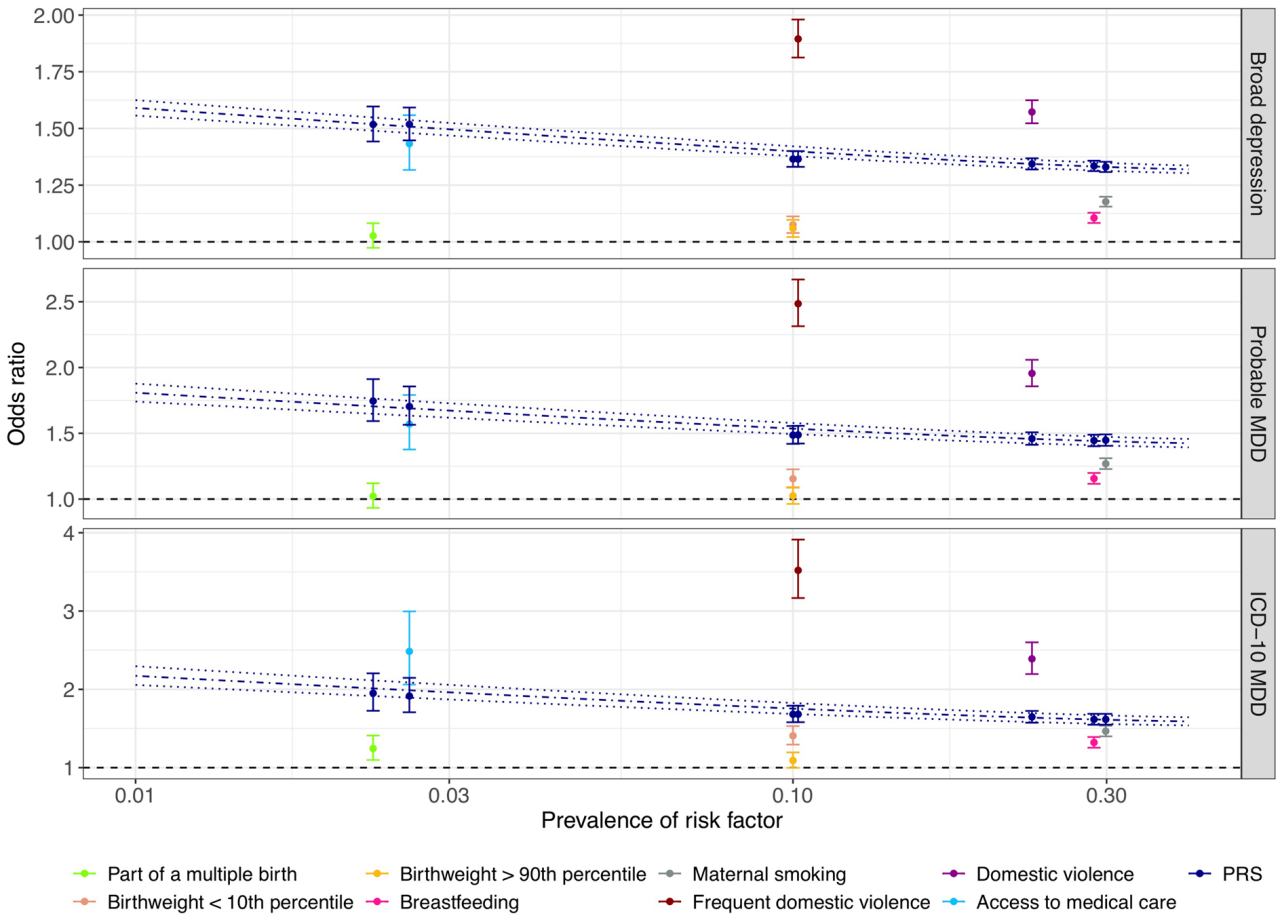
Comparison of associations between genetic and early life risk factors and depression phenotypes. The odds of depression associated with being exposed to each early life risk factor compared to the rest of the population are illustrated with respect to the prevalence of each risk factor. Individuals who were not breastfed as a baby and those who did not have access to medical care were considered to be at risk of depression. Odds ratios of depression were also derived by risk comparison between a high-risk group defined at a PRS cutoff corresponding to the prevalence of each early life risk factor and the rest of the test population, respectively, indicated in dark blue color. Error bars indicated 95% confidence intervals. Using the PRS as a continuous risk factor, predicted odds ratios with respect to the proportion of individuals considered to be at risk of depression are illustrated by the dark blue dashdotted line, with dark blue dotted lines indicating the 95% confidence interval. X-axis is on a logarithmic scale.

We found that the PRS and most of the early life risk factors under investigation were significantly associated with depression phenotypes in the test dataset (Figure 3; Supplementary Table S6). Importantly, a one SD increase in the PRS was associated with 1.19-fold increased odds of broad depression (95% CI: 1.18–1.20; value of *p* < 2.2 × 10^−308^; FDR < 2.2 × 10^−308^), 1.25-fold increased odds of probable MDD (95% CI: 1.23–1.26; value of *p* = 1.5 × 10^−208^; FDR = 1.4×10^−207^), and 1.33-fold increased odds of ICD-10 MDD (95% CI: 1.31–1.36; value of *p* = 6.5 × 10^−169^; FDR = 4.4 × 10^−168^; Figure 3; Supplementary Table S6). Among the early life risk factors, domestic violence demonstrated the strongest association with depression phenotypes. Compared to the rest of the population, 10.2% of the individuals who experienced frequent domestic violence had 1.89-fold increased odds of broad depression (95% CI: 1.81–1.98; value of *p* = 2.5 × 10^−176^; FDR = 2.0 × 10^−175^), 2.49-fold increased odds of probable MDD (95% CI: 2.31–2.67; value of *p* = 7.0×10^−138^; FDR = 3.4 × 10^−137^), and 3.52-fold increased odds of ICD-10 MDD (95% CI: 3.17–3.91; value of *p* = 4.5 × 10^−120^; FDR = 1.4 × 10^−119^; Figure 3; Supplementary Table S6).

Notably, the PRS was more predictive of the depression phenotypes than being part of a multiple birth, extreme birthweight, being breastfed as a child, and maternal smoking around birth, since high-risk groups defined based on the PRS cutoffs corresponding to risk factor prevalence were at a more evidently elevated level of risk of depression (section Methods; Figure 3; Supplementary Table S6). For example, maternal smoking around birth affected 29.9% of the individuals, conferring an odds ratio of 1.18 (95% CI: 1.16–1.20; value of *p* = 1.2 × 10^−66^; FDR = 2.8 × 10^−66^), 1.27 (95% CI: 1.23–1.31; value of *p* = 2.2 × 10^−47^; FDR = 3.7 × 10^−47^), and 1.47 (95% CI: 1.40–1.54; value of *p* = 5.5 × 10^−59^; FDR = 9.8 × 10^−59^) for broad depression, probable MDD, and ICD-10 MDD (Figure 3; Supplementary Table S6), respectively. In contrast, having a PRS among the highest 29.9% of the population was associated with 1.33-fold increased odds of broad depression (95% CI: 1.31–1.35; value of *p* = 5.5×10^−238^; FDR = 1.0 × 10^−236^), 1.45-fold increased odds of probable MDD (95% CI: 1.40–1.49; value of *p* = 3.0 × 10^−129^; FDR = 1.3 × 10^−128^), and 1.62-fold increased odds of ICD-10 MDD (95% CI: 1.55–1.69; value of *p* = 2.7 × 10^−108^; FDR = 7.5 × 10^−108^; Figure 3; Supplementary Table S6). Not having access to medical care appeared to have a similar predictive performance as the PRS (Figure 3; Supplementary Table S6).

### Joint modeling of genetic and early life risk factors achieved improved predictive performance

**Figure 4:**
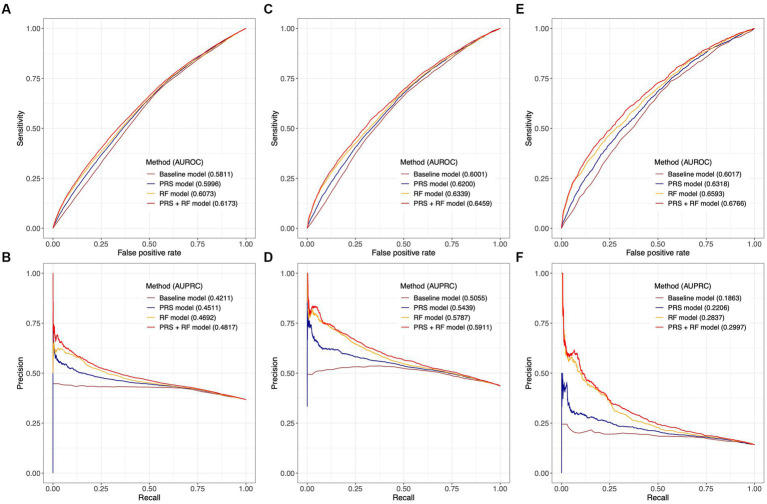
Comparison of predictive performance between joint prediction models. Receiver-operating characteristic curves and precision-recall curves are illustrated for broad depression in **(A,B)**, probable MDD in **(C,D)**, and ICD-10 MDD in **(E,F)**. AUROC and AUPRC are denoted for each model. The value of AUPRC depends on the model performance as well as the proportion of cases in the test population. Confidence intervals of AUROC and AUPRC based on 1,000 bootstrap replicates are illustrated in Supplementary Figure S3. All risk prediction models included age, age (Hawton et al., 2013), and sex as candidate predictors.

In the test dataset, all PRS + RF models demonstrated superior predictive performance compared to the RF models, PRS models, and baseline models. Specifically, the three PRS + RF models achieved the highest AUROC of 0.6173 (95% CI: 0.6129–0.6219) and AUPRC of 0.4817 (95% CI: 0.4752–0.4886) for predicting broad depression (Figures 4A,B), 0.6459 (95% CI: 0.6395–0.6522) and 0.5911 (95% CI: 0.5815–0.6010) for predicting probable MDD (Figures 4C,D), and 0.6766 (95% CI: 0.6633–0.6886) and 0.2997 (95% CI: 0.2815–0.3189) for predicting ICD-10 MDD (Figures 4E,F), respectively. Although the magnitudes of difference in AUROC and AUPRC between predictive models were not dramatic, the observed improvement in predictive performance was significant (all comparisons had a bootstrap value of *p* < 0.001 based on 1,000 bootstrap replicates; section Methods; Supplementary Figure S3). Notably, compared to other models, the PRS + RF models almost always achieved the highest sensitivity, specificity, and precision, when the same proportion of the population was considered at-risk (Figures 4A–F; Supplementary Table S7). The improved discriminative power of the PRS + RF models was also supported by consistently positive NRI at various cutoffs for identifying at-risk individuals and IDI (Supplementary Table S8).

## Discussion

In this study, leveraging the largest meta-analysis of MDD GWASs, we developed a PRS aggregating risk conferred by genetic variants throughout the genome. Based on a subset of the European ancestry population from the UK Biobank, we detected weak to moderate associations between the PRS and several early life risk factors. The PRS showed a significant association with depression phenotypes, with a magnitude of association similar to or larger than that of most early life risk factors alone under investigation, except for domestic violence. Furthermore, we constructed joint predictive models integrating genetic and early life risk factors which demonstrated improved predictive performance over more simplistic models, although the magnitude of performance improvement was limited.

Our results may have important clinical and research implications. On the one hand, findings of association tests reinforced the roles of key risk factors. Importantly, the PRS was associated with modifiable early life risk factors of high impact on adult depression, such as domestic violence, having no access to medical care, and maternal smoking around birth (Wiersma et al., 2009; Mandelli et al., 2015). Notably, these associations with parental behaviors among the children may reflect parental genetic associations with risk factors, demographic (such as assortative mating) effects, or indirect genetic effects via shared environment in large-scale GWASs (Robinson et al., 2017; Kong et al., 2018; Young et al., 2019; Howe et al., 2022; Lu et al., 2022c). To fully elucidate the sources of such genetic associations, studies focusing on disentangling parental genetic effects from the overall genetic effects, possibly through the use of non-transmitted alleles (Spielman and Ewens, 1996; Gauderman et al., 1999; Kong et al., 2018), as well as within-family GWASs (Howe et al., 2022) may be warranted. Nonetheless, these validated risk factors are strongly implicated as preventive and early intervention targets for reducing risk of depression among at-risk children.

On the other hand, despite strong correlation between risk factors, we showcased that stepwise regression implementing variable selection could effectively reduce the model redundancy of joint predictive models and achieve improved predictive performance. In particular, the PRS + RF model significantly outperformed the RF model and the PRS model with higher AUROC and AUPRC, and consistently demonstrated the highest sensitivity, specificity, and precision at different risk thresholds. However, it is worth noting that the increase in these performance metrics was marginal in magnitude, and the absolute accuracy of all models was limited. Therefore, we do not anticipate that the current models will have direct clinical utility. Nevertheless, we posit that our analytical framework may be generalized for better understanding of risk factors and improving risk prediction of complex diseases with a significant genetic component, including but not limited to psychiatric disorders.

Our study has two strengths. First, we adopted LDpred (Vilhjalmsson et al., 2015) to develop a PRS for a polygenic psychiatric disorder, which ensured that the PRS could capture a substantial proportion of the genetic liability while rigorously reducing bias due to LD. This PRS allowed accurate assessment of the association between the genetic predisposition and early life risk factors, and formed the basis of the joint predictive models. Second, we defined three depression phenotypes (Howard et al., 2018) to account for the high heterogeneity of depression while mitigating the effects of possible inconsistencies and errors in data collection and case definition. As expected, all risk factors demonstrated the strongest association with the most strictly defined ICD-10 MDD, while milder yet consistent associations with probable MDD and broad depression strongly supported the validity of our results.

Our study has important limitations. First of all, our analyses were restricted to individuals of European ancestry since the MDD GWASs were predominantly based on European ancestry individuals. It has been widely recognized that PRSs developed based on a European ancestry population can have substantially attenuated predictive performance in non-European ancestry populations due to discrepancies in LD structure, minor allele frequencies, effect sizes of causal variants (Martin et al., 2017, 2019b), etc. Moreover, our findings have not been replicated in other cohorts. Although the test dataset did not overlap with the model selection dataset, it is worth noting that familial relatedness is not uncommon in the UK Biobank (Bycroft et al., 2018). Therefore, the metrics of predictive performance obtained in this study are likely specific to the UK Biobank. We anticipate investigations in diverse populations will more thoroughly evaluate and improve the utility of genetic and early life risk factors in risk prediction of depression. Last, our study only considered a limited number of early life risk factors, whereas other known risk factors, such as socioeconomic status (Everson et al., 2002; Gilman et al., 2002; Goodman and Huang, 2002), substance abuse (Levy and Deykin, 1989; Goodman and Huang, 2002; Rao, 2006), and traumatic events (other than childhood domestic violence; Shalev et al., 1998; Young and Korszun, 2010; Mandelli et al., 2015), were not included due to unavailability of data or risk of reverse causation or confounding. Importantly, while we did not detect significant interaction effects between the PRS and selected early life risk factors in our study, existing studies have identified possible interaction effects between genetic and lifetime traumatic events on depression (Coleman et al., 2020; Chuong et al., 2022), although such interaction effects may be sensitive to model specification (Coleman et al., 2020). Our analyses examining interaction effects should also be viewed as exploratory, given the high complexity of the models and the potential incomplete consideration of confounding factors. More comprehensive modeling of additional risk factors and their interplay with the genetic predisposition while accounting for multicollinearity to improve model stability, and mitigating reverse causation and confounding effects may yield novel insights into the pathogenesis and progression of depression, and should be warranted in future studies.

In summary, we have developed a PRS consistently associated with depression phenotypes. This PRS was more or equally strongly associated depression phenotypes compared to most early life risk factors, except domestic violence. Despite partial dependence, genetic and early life risk factors should be explored jointly as tools to provide better risk stratification for identifying individuals at high risk of depression.

## Data availability statement

The original contributions presented in the study are included in the article/Supplementary material, further inquiries can be directed to the corresponding authors.

## Ethics statement

The studies involving human participants were reviewed and approved by North West Centre for Research Ethics Committee (11/NW/0382). The patients/participants provided their written informed consent to participate in this study.

## Author contributions

TL and CG designed the study. TL curated and managed data, performed the computational analyses, visualized the results and wrote the initial manuscript. TL, PS, and CG interpreted the results. All authors contributed to the article and approved the submitted version.

## Funding

This research has been conducted using the UK Biobank resource under Application Number 60755. This study was enabled in part by support provided by Calcul Québec and Compute Canada. TL has been supported by a Schmidt AI in Science Postdoctoral Fellowship, a Vanier Canada Graduate Scholarship, and a Fonds de Recherche du Québec - Santé Doctoral Training Fellowship. CMTG is supported by a Canadian Institutes of Health Research grant (PJT-148620).

## Conflict of interest

TL was an employee of 5 Prime Sciences Inc.

The remaining authors declare that the research was conducted in the absence of any commercial or financial relationships that could be construed as a potential conflict of interest.

## Publisher’s note

All claims expressed in this article are solely those of the authors and do not necessarily represent those of their affiliated organizations, or those of the publisher, the editors and the reviewers. Any product that may be evaluated in this article, or claim that may be made by its manufacturer, is not guaranteed or endorsed by the publisher.
