# Supplementary material for: Development of risk prediction models for depression combining genetic and early life risk factors
Source: Front Neurosci. 2023 Jul 18;17:1143496. doi: 10.3389/fnins.2023.1143496 (PMC10390723; doi:10.3389/fnins.2023.1143496)
Supplement: Supplementary file 1 [file Data_Sheet_1.PDF]

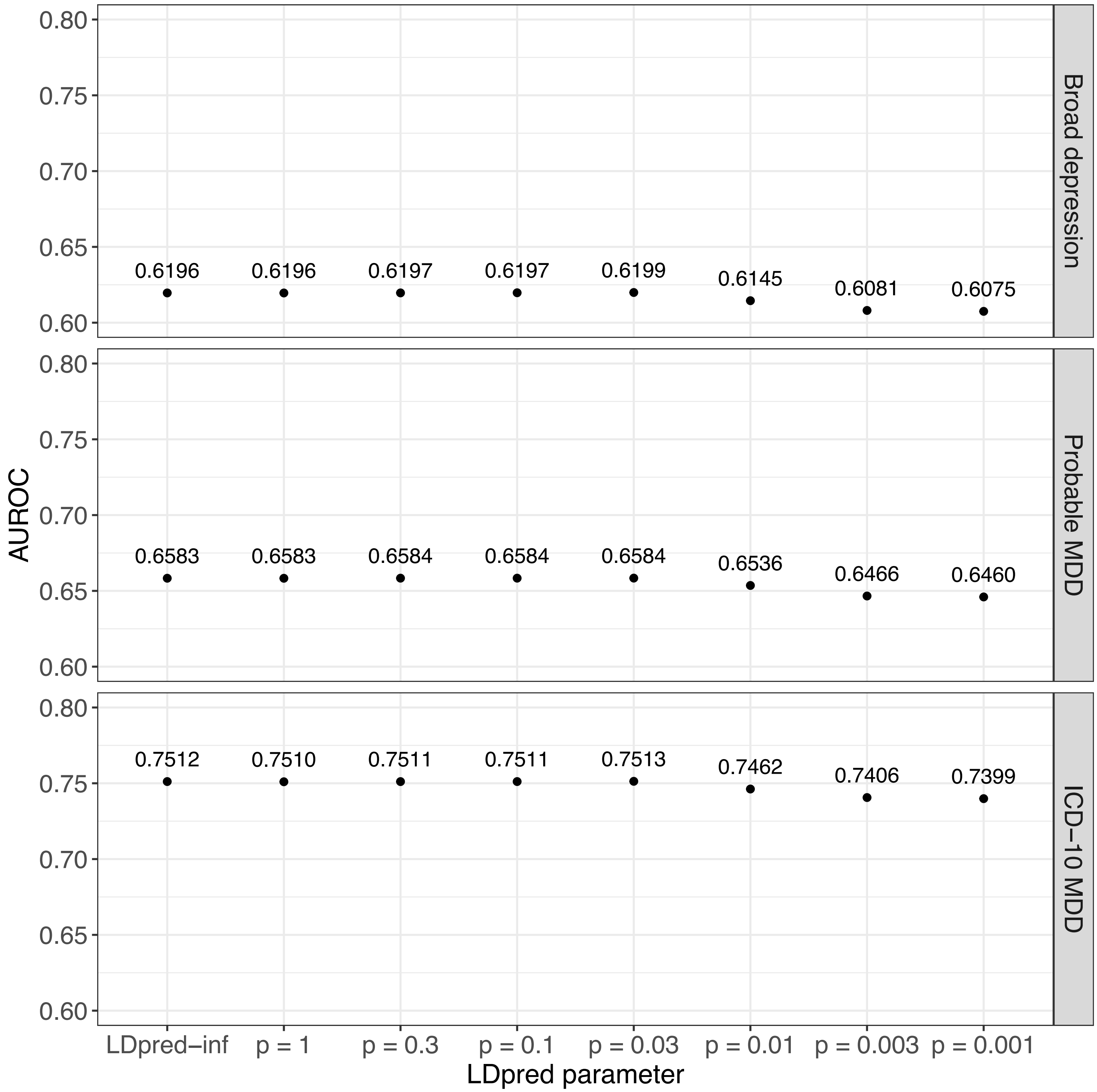

Figure S1. Optimization of LDpred parameter in the UK Biobank model selection dataset. For all three depression phenotypes, p = 0.03 was selected as the parameter resulting in the best-performing PRS.

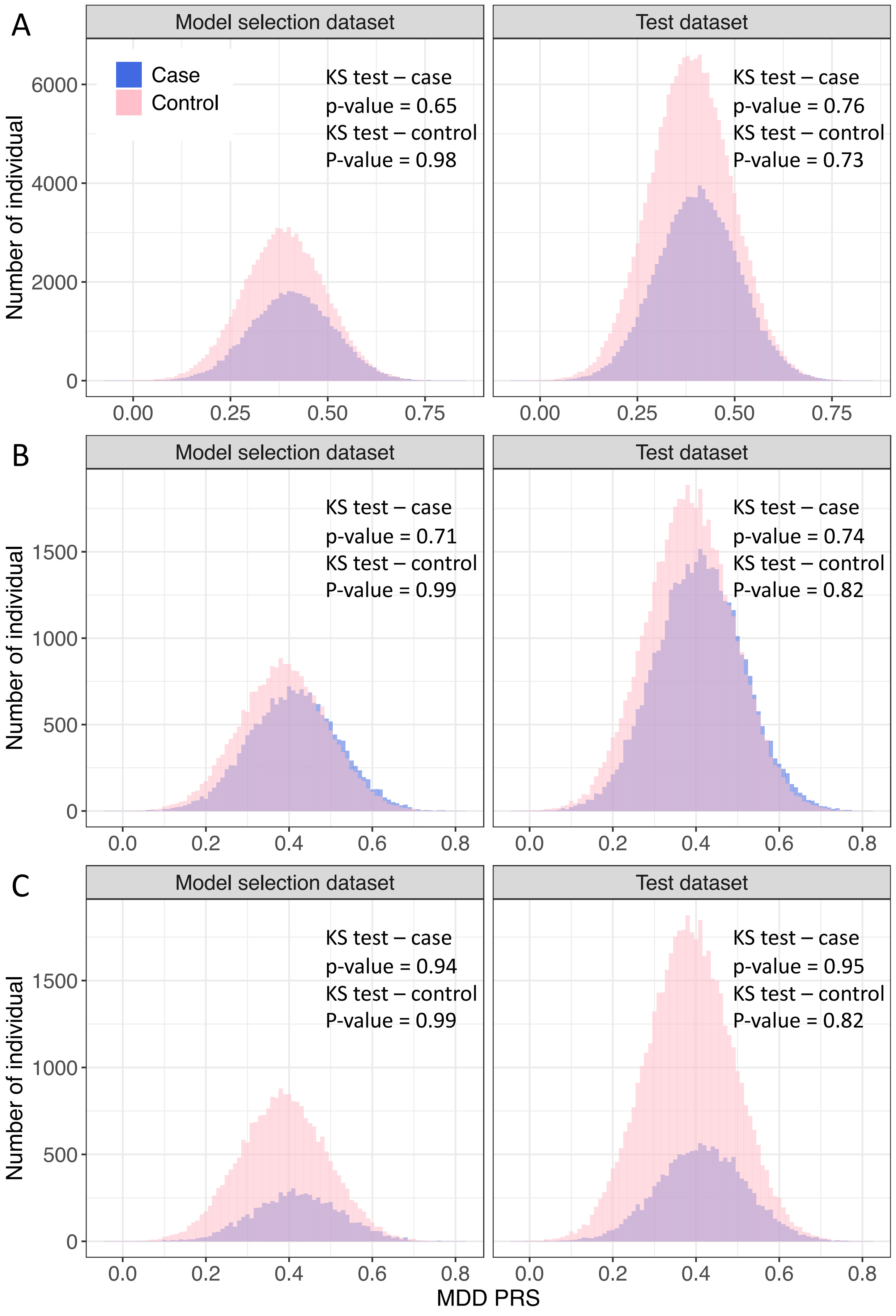

Figure S2. Normality of PRS in the UK Biobank. Distributions of the PRS are illustrated for (A) cases of broad depression and controls, (B) cases of probable MDD and controls, and (C) cases of ICD-10 MDD and controls, separately in the model selection dataset and the test dataset. Kolmogorov-Smirnov (KS) test p-values are denoted.

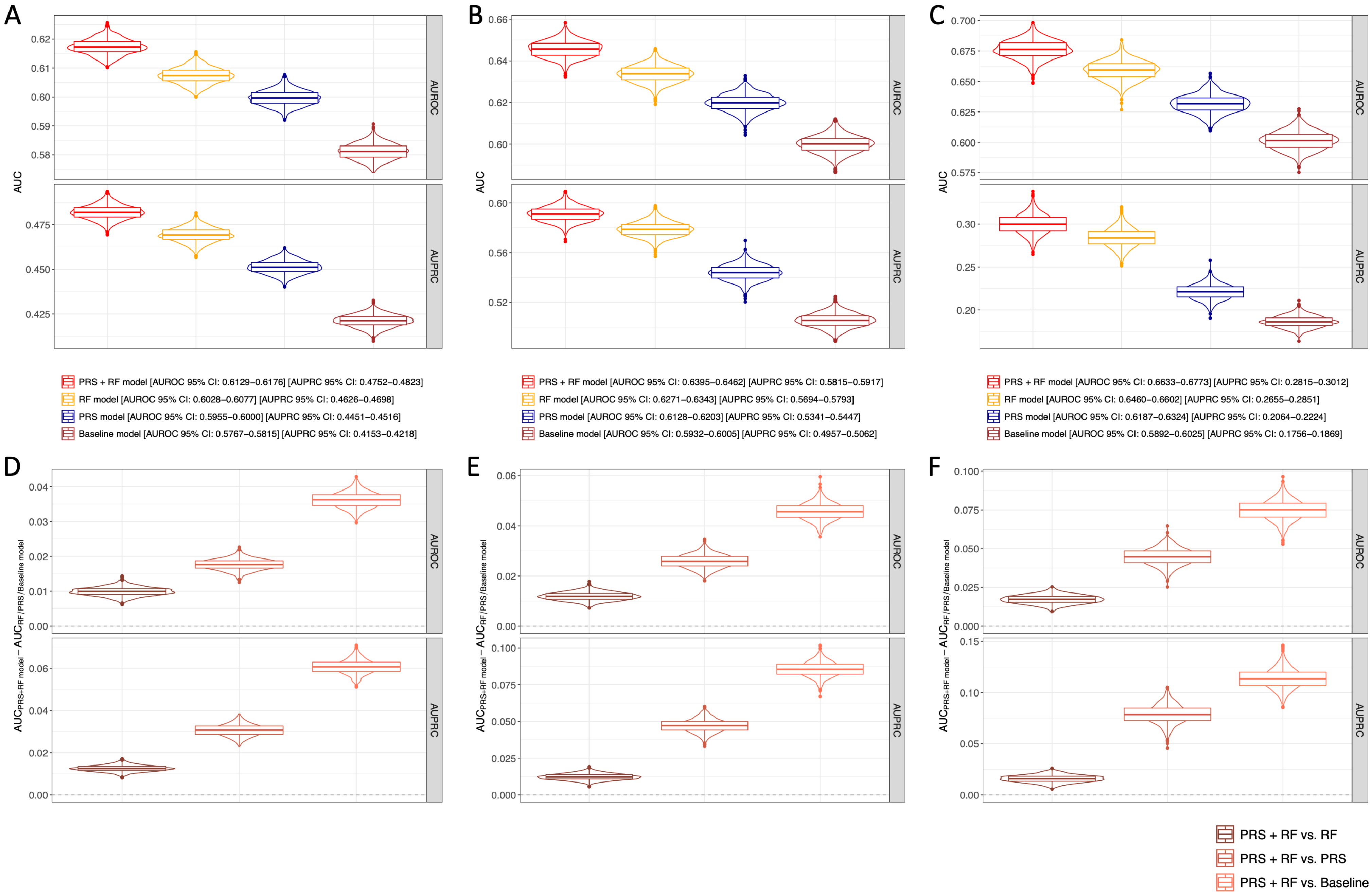

Figure S3. Comparison of predictive performance between joint prediction models by 1000 bootstrap replicates. Distributions of area under the receiver-operating characteristic curve (AUROC) and area under the precision-recall curve (AUPRC) are illustrated by box plots and violin plots for (A) broad depression, (B) probable MDD, and (C) ICD-10 MDD. In each bootstrap replicate, the difference between the PRS+RF model and the other three predictive models ($AUC_{PRS+RF\ model}$-$AUC_{RF/PRS/Baseline\ model}$) was recorded. Distributions of such differences across 1000 bootstrap replicates are illustrated by box plots and violin plots for (A) broad depression, (B) probable MDD, and (C) ICD-10 MDD. Since the PRS+RF model always had the highest AUROC and AUPRC in all bootstrap replicates, all comparisons had a bootstrap p-value < 0.001. Each rectangular box indicates the interquartile range with a horizontal line denoting the median. Whiskers extending from the box represent a maximum of 1.5 times the interquartile range from the upper and lower quartiles. Individual dots represent outliers. 95% confidence intervals were obtained based on 1000 bootstrap replicates.
